# Supplementary material for: Spatial mapping of mobile genetic elements and their bacterial hosts in complex microbiomes
Source: Nat Microbiol. 2024 Jun 25;9(9):2262–77. doi: 10.1038/s41564-024-01735-5 (PMC11371653; doi:10.1038/s41564-024-01735-5)
Supplement: Supplementary file 1 — Reporting Summary [file 41564_2024_1735_MOESM1_ESM.pdf]

nature portfolio

Corresponding author(s): Iwijn De Vlaminck

Last updated by author(s): 29 Apr 2024

Reporting Summary

Nature Portfolio wishes to improve the reproducibility of the work that we publish. This form provides structure for consistency and transparency in reporting. For further information on Nature Portfolio policies, see our Editorial Policies and the Editorial Policy Checklist.

Statistics

For all statistical analyses, confirm that the following items are present in the figure legend, table legend, main text, or Methods section.

n/a	Confirmed	
☐	☒	The exact sample size (*n*) for each experimental group/condition, given as a discrete number and unit of measurement
☐	☒	A statement on whether measurements were taken from distinct samples or whether the same sample was measured repeatedly
☐	☒	The statistical test(s) used AND whether they are one- or two-sided<br>*Only common tests should be described solely by name; describe more complex techniques in the Methods section.*
☒	☐	A description of all covariates tested
☐	☒	A description of any assumptions or corrections, such as tests of normality and adjustment for multiple comparisons
☐	☒	A full description of the statistical parameters including central tendency (e.g. means) or other basic estimates (e.g. regression coefficient) AND variation (e.g. standard deviation) or associated estimates of uncertainty (e.g. confidence intervals)
☐	☒	For null hypothesis testing, the test statistic (e.g. F, t, r) with confidence intervals, effect sizes, degrees of freedom and P value noted<br>*Give P values as exact values whenever suitable.*
☒	☐	For Bayesian analysis, information on the choice of priors and Markov chain Monte Carlo settings
☒	☐	For hierarchical and complex designs, identification of the appropriate level for tests and full reporting of outcomes
☒	☐	Estimates of effect sizes (e.g. Cohen's *d*, Pearson's *r*), indicating how they were calculated

Our web collection on statistics for biologists contains articles on many of the points above.

Software and code

Policy information about availability of computer code

Data collection

Confocal microscope images were collected on Zeiss LSM i880 with Zen 2.3 SP1 FP3 (Black) v14.0.28.201 software. Nanopore reads were collected on MinION Mk1B with MinKNOW v23.07.15.

Data analysis

DNA-FISH Split-Probe design. Probes were designed using a custom Snakemake v7.18.2 pipeline with rules written in Python v3.6.8 using numpy v1.15.4 and pandas v0.24.1.53, Target gene sequences were taken as inputs along with a reference blast database. The target was aligned to the blast database and all significant alignments were recorded for future filtering (blastn v2.13.0). All possible oligonucleotide probes were designed to be complementary to the coding strand of the target gene (i.e. the same sense as the mRNA) using Primer3 v2.3.5.55 Pairs of Probes in this pool were identified as any probes aligning less than three base pairs distant from each other. These probe pairs were then blasted against the reference database using blastn from NCBI. On-target blast results were removed from the results using the target gene alignment IDs. Non-significant blast results were then filtered using user-defined parameters. These include maximum continuous homology (12), GC count (7), and melting temperature (46℃). All blast results with values in these parameters that were less than the specified thresholds were removed as “non-significant alignments”. The remaining blast results were considered “significant” or likely to produce off-target signal. Probe pairs were removed when both probes had off-target homologies to nearby regions in the reference database. This nearness parameter is another user-defined threshold. The remaining probe pairs were then sorted with favored probes having low levels of off-target homology. Going down the sorted list, probe pairs were then selected to tile along the gene without overlapping. Selected probes were then appended with appropriate flanking regions so that the target would be stained with the intended fluorophore (Supp. Tab. 1). Two base-pair spacers nucleotides between the flanking region and the probe were selected to minimize the off-target homology of the full-length probes in a similar manner to how probe pairs were sorted by blast results. The pool of selected probe pairs was then evaluated by searching for any off-target homologies where two probes were nearby each other. “Helper” probes were then selected from the Primer3 to tile along the gene without overlapping the existing probes. The final probes were then submitted for oligo synthesis to Integrated DNA Technologies (IDT) at a concentration of 200µM.

DNA-FISH single probe design. Single probes were designed much as the split probes up to the Primer3 step. Then, instead of pairing probes, the probes were all blasted against the database and the blast results were filtered as the split probes were for "significant" off-target homologies. Probes with any significant off-target homologies were removed and the remaining probes were tiled along the target gene to ensure no overlap. The selected probes were then paired with flanking regions for the readout stain and two base pair spacers were added and optimized as in the split probe design. The resulting probes were submitted for synthesis to IDT.

Orthogonal probe design. Probes with zero significant off-target blasts were selected from split probe pairs for different genes. For example if the left probe from a pair targeting Gene A has zero off-target blasts it is selected, then the right probe from a pair targeting Gene B is selected. The concept is that it is very unlikely these probes will hybridize close enough to each other to initiate HCR fluorescence amplification. Three right probes and three left probes

Manual spot background filtering. Images were processed using a combination of Python scripts using numpy v1.21.2 and interactive Jupyter notebooks v1.0.0 to iteratively adjust and check the results of parameter adjustments. We first applied deconvolution and pixel reassignment to Airyscan images to return a super resolution image using Zen 2.3 SP1 FP3 (Black) v14.0.28.201. Taking this as input, we then set a manual threshold to identify the foreground. We set the threshold such that visually distinct spots were mostly masked as separate objects. For images with high levels of non-specific signal, "blobs", we used watershed segmentation with the background thresholded image as seed and a low intensity background thresholded image as a mask. We measured the foreground objects using skimage functions. We then removed objects larger than the threshold area. Here we set the threshold such that objects containing 1-3 neighboring spots were not removed, but objects with the continuous high signal indicative of non-specific binding were removed. We then filtered the remaining objects based on maximum intensity. Here we set the threshold to remove objects with continuous low intensity, but keep objects with high intensity peaks.

Semi-automated image segmentation. For batches of images, an example image was selected and a zoom region within the image was selected to manually adjust segmentation parameters. In Airyscan images, segmentation parameters were set separately for cell and spot channels. In spectral images, the channels were aligned using phase cross correlation to correct for drift while switching between lasers, then the maximum projection or sum projection along the channel axis was used for segmentation. The image background mask was determined by applying a manual threshold, loading a manually adjusted background mask (as in some spot segmentation), or k-means clustering of pixel intensities. For segmentation pre-processing, images were optionally log normalized to enhance dim cells, then denoised using Chambolle total variation denoising implemented in skimage with adjustments to the weight parameter. In airyscan images it was sometimes necessary to blur subcellular features, so a gaussian filter could be applied with adjustments to the sigma parameter. If objects were densely packed and edge enhancement was required, we applied the local neighborhood enhancement algorithm to generate an edge-enhanced mask.8 In certain cases, difference of gaussians was also used for edge enhancement of the preprocessed image. We then used the watershed algorithm with peak local maxima as seeds to generate the final segmentation. Once the parameters were set, a Snakemake pipeline applied the segmentation parameters to all images in the batch. Segmented objects were measured using standard skimage functions. For spot images, local maxima were determined using skimage functions and objects with multiple local maxima were split into new objects using Pysal60 to generate a Voronoi diagram from the maxima to set borders between the new objects. Spots were assigned to cells based on object overlap or by radial distance between centroids.

Manual Cell and spot counting. In the 30 minute and 40 minute timepoints of the phage infection, many of the infected cells had reduced 16S rRNA signal and lysed cells had caused clumps of cells to form that were difficult to segment. To count cells and classify them by their number of phage spots we used a manual counting strategy where each image was loaded into a graphic design tool (Affinity Designer) and cells of each type were counted and marked by hand. We counted a minimum of 1000 cells for each time-MOI combination.

AMR and prophage gene discovery. Raw reads were processed with PRINSEQ lite v0.20.4 and trimmomatic v0.36 to remove optical duplicates and sequencing adapters. Reads mapping to the human genome were discarded using BMTagger. Clean reads were assembled using SPAdes v3.14.0 (paired-end mode and –meta option) and reads were aligned to contigs using minimap2 v2.17. Contigs were resolved into metagenomic bins using vamb v3.0.2 with reduced hyperparameters (-l 24, -n 384 384). Completeness and contamination of bins were evaluated with checkM v1.1.2, and taxonomies were assigned to bins using GTDB-Tk v1.0.2 with GTDB release 207. Read-level taxonomic relative abundance estimates were carried out with Kraken2 v2.1.2 and Bracken v2.6.1. Lytic and lysogenic phage were identified and evaluated for induction using VIBRANT v1.2.1 and PropagAtE v1.0.0, requiring a minimum length of 5000 bp and at least 10 ORFs per scaffold. Antibiotic resistance genes were annotated on contigs and mobile elements using Resistance Gene Identifier v5.2.0 against the CARD database v3.1.0 supplemented with the Resistomes & Variants dataset v3.0.8.

Plasmid prediction. Long raw data was processed using Dorado v0.4.2. Long reads were assembled using Flye v2.9.2. Hybrid metagenomic assembly was performed using OPERA-MS on clean short reads and Dorado duplex outputs for long reads. Plasmids were predicted using geNomad v1.7.1. Putative plasmids from the hybrid assembly were identified in the long read-only assembly to help with circularization of the sequence. Short reads were aligned to putative plasmids assemblies using bowtie2 v2.5.1. Long reads were aligned using bwa mem v0.7.17 with Nanopore parameters (-x ont2d) and filtered to remove short partial alignments (identity > 80%, query coverage > 80%). Coverage measurements were done with samtools v1.18. GC skew was calculated as (G50bp – C50bp)/(G50bp + C50bp) where G50bp and C50bp are the number of G and C bases in a 50bp window, and the location of OriC was estimated visually based on GC skew plotting. The number of GGGG and CCCC stretches in a plasmid sequence was counted as a 4bp window at each base; for example, GGGGG results in two counts.

Spatial autocorrelation analysis. A neighbor spatial connectivity matrix was constructed from cell segmentation centroids using a Voronoi diagram algorithm from Pysal Each cell was given a binary mark indicating presence of MGE spot. The weight matrix and marked cells were used in a global Moran's I test from Pysal to calculate spot autocorrelation. The measured Moran's I value was compared against a simulation based null model that spots are randomly distributed within the cell space. P-values were calculated using a two tailed Monte Carlo test.

Large scale spot density plots. After spot segmentation, the universal 16S rRNA signal was used to create a global mask to identify the foreground. For each pixel in the foreground, we used the nearest neighbors algorithm to calculate the number of spots within a certain radius of each grid point, and divided by the area of the search to get a density value for each point.

Spatial association measurements. We performed two versions of spot colocalization. First in a given color channel, for each spot we used the nearest neighbors algorithm to determine whether there were spots of the other color(s) within a 0.5µm radius and calculated the fraction of spots colocalized with each of the other colors based on the number of spots in the reference channel. We repeated the measurement for each color channel. In the second version, we overlaid the spots from each channel (labeled as different spot types), divided the image into a grid of squares with 5µm edges, classified each square based on the number of spot types present, counted the number of squares of each

type, and normalized by the total number of squares with at least one spot type.

AMR gene distribution measurements. Segmented spots were converted into a point pattern object in the PySAL python package. Simulations were generated using the PoissonPointProcess function to generate 100 realizations of the point pattern. Nearest neighbor distances were generated from these objects with the nnd function. Histograms values were calculated using 1µm bins. The cumulative distribution G(d) was calculated using the G function from PySAL. The pair correlation function R(d) was calculated using the K function from PySAL.

Genus level probe design. We performed full length 16S rRNA sequencing and taxonomic classification as previously described8 on the extracted DNA used for metagenomic sequencing in DNA Extraction. We searched for previously designed genus level FISH probe sequences29 and blasted the probes against our full length 16S rRNA data using blastn. We filtered results to remove “non-significant” alignments as defined above in DNA-FISH Split-Probe design, determined the fraction of significant alignments to non-target genera, and removed probes with off-target rate greater than 0.1. We then selected 5-bit binary barcodes for each genus to maximize the distance between barcode fluorescent spectra. Distance between sum-normalized arrays of reference spectra was calculated using a “e uclidean distance of cumulative spectrum” metric.77 Based on the binary barcodes we concatenated a readout sequence to the three prime end of each probe sequence such that the readout sequence would hybridize the appropriate fluorescent readout probe for the barcode (Supp. Tab. 9). For barcodes with multiple colors in the barcode, we created separate probes concatenated with each readout sequence. We created barcodes that used only the 488 nm, 514 nm, and 561 nm lasers, thus reserving the 633 nm laser for MGE-FISH and the 405 nm laser for the universal EUB338 16S rRNA stain. For stains where we targeted only 5 genera, we simply used a different fluorophore for each genus probe.

Pixel-level spectral classification. To classify pixels in the 5 genus experiment (e.g. Fig. 3c), we aligned the laser channels of the spectral images using phase cross correlation, then we performed gaussian blurring (sigma=3) on each spectral channel to reduce the noise in each pixel’s spectra. We acquired a maximum intensity projection along the channel axis, selected a background threshold, and generated a mask. To account for nonspecific binding, which generates a low intensity background signal with the “11111” (all 5 fluorophores) spectral barcode, we multiplied the “11111” reference spectrum by a scalar and subtracted the scaled spectrum from each pixel’s measured spectrum (reference spectra for each barcode were collected as previously described). We visualized the pixel spectra before and after subtraction and adjusted the scalar such that the visually apparent background was removed (scalar=0.05). The adjusted pixel spectra were stored in a “pixel spectra matrix” with the following shape: (number of pixels,number of spectral channels). The reference spectra for all barcodes were sum normalized and merged in a “reference spectra matrix” with the following shape: (number of spectral channels,number of barcodes). We performed matrix multiplication between the “pixel spectra matrix” and the “reference spectra matrix” to get a “classification matrix” with shape: (number of pixels,number of barcodes). Separately, we evaluated the reference spectra and created a boolean array indicating whether or not we expected a signal from each of the three lasers. We merged these arrays into a “reference laser presence” matrix with shape: (number of lasers,number of barcodes). Then, for each adjusted pixel spectrum we measured the maximum value for each laser, normalized these values by the highest of the three values, and set minimum threshold values (threshold488=0.3, threshold514=0.4, threshold561=0.3) to create a “pixel laser presence” boolean matrix with shape: (number of pixels,number of lasers). We performed matrix multiplication between the “pixel laser presence” matrix and the “reference laser presence” matrix to get a matrix with shape: (number of pixels,number of barcodes). We performed element-wise multiplication between this matrix and the “classification matrix” to remove barcodes from the classification matrix if the signal from one of the lasers was too low. For each pixel, we selected the barcode with the highest value in the adjusted “classification matrix”.

Cell segmentation level spectral classification. We aligned the laser channels using phase cross correlation, then applied the Semi-automated image segmentation method to the maximum projection of the spectral channels. In the 5-genus experiment, for each object in the cell segmentation, if all the pixels within the object were assigned to the same taxon, we assigned that taxon to the object. If multiple taxa were represented in the cell pixels, the object was split into multiple new objects such that each new object encompassed pixels of only one taxon. To classify segmented cells in the 18-genus experiment (e.g. Fig. 3g), We acquired the mean spectrum of pixels within each segmented object, then calculated the pairwise cosine distances between all mean cell spectra and clustered the spectra into 20 groups using agglomerative clustering. We then manually classified each cluster by visually comparing them to pure reference spectra, which we acquired as reported previously.

Registration of Airyscan and Lambda mode images. Since HiPR-FISH images were captured using Lambda mode for spectral measurement and MGE-FISH images were captured using Airyscan mode for improved resolution, we rescaled the HiPR-FISH images so that the pixel size matched the MGE-FISH images. We used phase cross-correlation to register shifts between the Airyscan 16s rRNA signal and the HiPR-FISH maximum projection image. We then applied these shifts to the Airyscan MGE-FISH images.

Taxon-spot spatial association measurements. We created isolated the a subset set of the cell centroids for each taxon. Then for each taxon we used the nearest neighbor algorithm to measure the distance from each spot to the nearest cell of that taxon and counted the number of spots where distance was less than 0.5µm. To calculate the fraction of spots and taxon cells, we divided the count by the total number of spots and total number of taxon cells respectively.

Random simulation of spot distribution. We used the foreground mask to create a list of pixel coordinates within the plaque cells, then used a random integer generator to select pixels by their list index. We used the randomly selected pixel coordinates as simulated spots and counted taxon-spot spatial associations as described above. This was repeated for 1000 simulations and we calculated the mean and standard deviation for the count values for each taxon. We then calculated the z-score for the count values: z=(count - mean) / standard deviation. P-values were calculated by counting the fraction of simulations with greater values than the observed value.

Statistics. Python v3.8.5 was used to generate statistics. Box plots consist of a bottom line representing the lower quartile (Q1), a line inside the box representing the median (Q2), a top line representing the upper quartile (Q3), an upper whisker extending from the top of the box indicating the maximum value within 1.5 times the interquartile range (IQR) above Q3, and a lower whisker extending from the bottom of the box indicating the minimum value within 1.5 times the IQR below Q1. Monte Carlo methods with 100 or 1000 simulations were used in two-sided tests to evaluate null hypotheses of random distribution of spots.

The specific implementation of code to generate figures presented here is available on GitHub at https://github.com/benjamingrodner/hipr_mge_fish (v1.0.0, https://zenodo.org/doi/10.5281/zenodo.11085744). The generalized pipeline for segmentation is available at https://github.com/benjamingrodner/pipeline_segmentation (v1.0.0, https://doi.org/10.5281/zenodo.11085837), while the generalized implementation of probe design is available at https://github.com/benjamingrodner/FISH_split_probe_design (v1.0.0, https://

doi.org/10.5281/zenodo.11085839).

For manuscripts utilizing custom algorithms or software that are central to the research but not yet described in published literature, software must be made available to editors and reviewers. We strongly encourage code deposition in a community repository (e.g. GitHub). See the Nature Portfolio guidelines for submitting code & software for further information.

Data

Policy information about availability of data

All manuscripts must include a data availability statement. This statement should provide the following information, where applicable:

- Accession codes, unique identifiers, or web links for publicly available datasets
- A description of any restrictions on data availability
- For clinical datasets or third party data, please ensure that the statement adheres to our policy

Illumina and PacBIO sequencing data are available at the NCBI Sequence Read Archive (SRA) with accession number PRJNA981198. Microscopy data have been deposited to Zenodo at https://doi.org/10.5281/zenodo.8015720 (Fig. 1b, Fig. S1a,b), https://doi.org/10.5281/zenodo.8015754 (Fig. 1c, Extended Data Fig. 1c,d, including count tables), https://doi.org/10.5281/zenodo.8015832 (Fig. 2, Extended Data Fig. 2, 3), https://zenodo.org/doi/10.5281/zenodo.11039333 (Fig. 3, Extended Data Fig. 4, including plasmid assembly with Illumina and Nanopore reads), and https://zenodo.org/doi/10.5281/zenodo.11039443 (Fig. 4, Extended Data Fig. 5, including plasmid assembly with Illumina and Nanopore reads).

GTDB release 207 is available at https://data.gtdb.ecogenomic.org/releases/release207/, CARD v3.1.0 is available at https://card.mcmaster.ca/download, PLSDB v.2023_11_03 is available at https://ccb-microbe.cs.uni-saarland.de/plsdb/plasmids/download/, RefSeq release 220 is available at https://ftp.ncbi.nlm.nih.gov/refseq/release/release-catalog/archive/, checkM database v2015-01-16 is available at https://zenodo.org/doi/10.5281/zenodo.7401544, geNomad database v1.7 is available at https://doi.org/10.5281/zenodo.10594875, and the Bakta database v5.0 is at https://doi.org/10.5281/zenodo.7669534. For VIBRANT, Pfam v32.0 is available https://ftp.ebi.ac.uk/pub/databases/Pfam/releases/Pfam32.0/, VOG v94 is available at https://fileshare.lisc.univie.ac.at/vog/vog94/, and KEGG v2019-03-20 is available at ftp://ftp.genome.jp/pub/db/kofam/archives/2019-03-20/.

Research involving human participants, their data, or biological material

Policy information about studies with human participants or human data. See also policy information about sex, gender (identity/presentation), and sexual orientation and race, ethnicity and racism.

Reporting on sex and gender	Sex and gender were not relevant in study design.
Reporting on race, ethnicity, or other socially relevant groupings	Socially relevant groupings were not relevant in study design.
Population characteristics	No covariate relevant population characteristics were considered in study design.
Recruitment	Two healthy volunteers donated samples were recruited Cornell. At Harvard School of Dental Medicine, one donor was recruited from patients in the advanced graduate periodontal department during their initial examination and/or dental hygiene therapy visit with inclusion criteria of age greater than 18 years and diagnosed periodontitis and exclusion criteria of greater than 20 cigarettes a day, antibiotic use within the last 8 weeks, and systemic condition requiring antibiotic prophylaxis.
Ethics oversight	The protocol for volunteer sample collection was approved by the Cornell Institutional Review Board (IRB) #2102010112. At Harvard School of Dental Medicine, IRB approval (IRB21-0662) was obtained for collection of patient specimens in the advanced graduate periodontal department.

Note that full information on the approval of the study protocol must also be provided in the manuscript.

Field-specific reporting

Please select the one below that is the best fit for your research. If you are not sure, read the appropriate sections before making your selection.

☒ Life sciences ☐ Behavioural & social sciences ☐ Ecological, evolutionary & environmental sciences

For a reference copy of the document with all sections, see nature.com/documents/nr-reporting-summary-flat.pdf

Life sciences study design

All studies must disclose on these points even when the disclosure is negative.

Sample size	No statistical methods were used to pre-determine sample sizes but our sample sizes are similar to those reported in previous publications. For cultured cell experiments, the number of cells measured was routinely in the thousands. Sample size was chosen based on fields of view, where each condition was measured with three tile scans composed of four fields of view each thus measuring thousands of cells. For oral plaque experiments, the target genes were unique to each volunteer, so multiple samples were not possible for a given target gene. Sample size was chosen based on fields of view, where each sample was measured with at least three tile scans composed of at least 9 fields of view each thus measuring thousands of cells.

Data exclusions	No data were excluded from the analysis.
Replication	All attempts at replication were successful. Multiple fields of view were collected for each sample. For oral plaque experiments, generally two technical replicates were performed. For cultured cell imaging, two replicates were performed.
Randomization	For technical controls, samples of cultured cells and plaque were allocated randomly.
Blinding	Blinding was not applicable in the methods development studies using cultured cells since imaging and image analysis settings were quantitatively standardized and replicated for negative and positive controls. Blinding was not needed for the descriptive and exploratory studies using plaque samples. No specific hypothesis was tested.

Reporting for specific materials, systems and methods

We require information from authors about some types of materials, experimental systems and methods used in many studies. Here, indicate whether each material, system or method listed is relevant to your study. If you are not sure if a list item applies to your research, read the appropriate section before selecting a response.

Materials & experimental systems

n/a	Involved in the study
☒	☐ Antibodies
☒	☐ Eukaryotic cell lines
☒	☐ Palaeontology and archaeology
☒	☐ Animals and other organisms
☒	☐ Clinical data
☒	☐ Dual use research of concern
☒	☐ Plants

Methods

n/a	Involved in the study
☒	☐ ChIP-seq
☒	☐ Flow cytometry
☒	☐ MRI-based neuroimaging
